# Quantifying the effectiveness of shoreline armoring removal on coastal biota of Puget Sound

**DOI:** 10.7717/peerj.4275

**Published:** 2018-02-23

**Authors:** Timothy S. Lee, Jason D. Toft, Jeffery R. Cordell, Megan N. Dethier, Jeffrey W. Adams, Ryan P. Kelly

**Affiliations:** 1 Department of Biology, East Carolina University, Greenville, NC, USA; 2 School of Marine and Environmental Affairs, University of Washington, Seattle, WA, USA; 3 School of Aquatic and Fishery Sciences, University of Washington, Seattle, WA, USA; 4 Department of Biology, Friday Harbor Laboratories, University of Washington, Friday Harbor, WA, USA; 5 Washington Sea Grant, College of the Environment, University of Washington, Seattle, WA, USA

**Keywords:** Shoreline, Armoring, Macroinvertebrates, Restoration, Effect size, Coastlines, Biota, Response, Cohen’s *D*, Restoration trajectory

## Abstract

Shoreline armoring is prevalent around the world with unprecedented human population growth and urbanization along coastal habitats. Armoring structures, such as riprap and bulkheads, that are built to prevent beach erosion and protect coastal infrastructure from storms and flooding can cause deterioration of habitats for migratory fish species, disrupt aquatic–terrestrial connectivity, and reduce overall coastal ecosystem health. Relative to armored shorelines, natural shorelines retain valuable habitats for macroinvertebrates and other coastal biota. One question is whether the impacts of armoring are reversible, allowing restoration via armoring removal and related actions of sediment nourishment and replanting of native riparian vegetation. Armoring removal is targeted as a viable option for restoring some habitat functions, but few assessments of coastal biota response exist. Here, we use opportunistic sampling of pre- and post-restoration data for five biotic measures (wrack % cover, saltmarsh % cover, number of logs, and macroinvertebrate abundance and richness) from a set of six restored sites in Puget Sound, WA, USA. This broad suite of ecosystem metrics responded strongly and positively to armor removal, and these results were evident after less than one year. Restoration responses remained positive and statistically significant across different shoreline elevations and temporal trajectories. This analysis shows that removing shoreline armoring is effective for restoration projects aimed at improving the health and productivity of coastal ecosystems, and these results may be widely applicable.

## Introduction

Worldwide, shorelines adjacent to bodies of fresh and salt waters face faster urbanization and population growth than other geographic regions (Neumann et al., 2015). Coastal regions have always experienced high immigration rates because of their ease of access to domestic and international shipping, military and defense uses, tourism, access to recreational activities, access to valuable ecosystem services, and employment opportunities (Bulleri, Chapman & Underwood, 2005; MEA, 2005; Gittman et al., 2015; Neumann et al., 2015). About 50% of the world’s population lives within 200 km from the coastlines and half of the world’s major city centers are located within 50 km from coasts (Stegeman & Solow, 2002; MEA, 2005). Many of these heavily populated coastal regions are in low-lying elevations. In 2000 these low-elevation coastal zones included nearly 11% of the world’s total coastal population, but by 2060 it is estimated that the population in these low-elevation coastal zones will be as great as 1.4 billion, or 12% of the world’s population (Neumann et al., 2015).

Coastal infrastructure and urban centers are exposed to various hazards including storms, large waves, flooding, sea level rise, and erosion (Jones & Hanna, 2004; McGranahan, Balk & Anderson, 2007). As a response, many coastal communities have established hardened structures such as bulkheads, jetties, riprap revetments and seawalls, a practice commonly called “shoreline armoring” that is part of ocean sprawl (Chapman, 2003; Bulleri & Chapman, 2010; Chapman & Underwood, 2011; Heerhartz et al., 2014; Gittman et al., 2015; Firth et al., 2016b). In some large urban centers such as San Diego Bay, Chesapeake Bay, Sydney Harbor, and Hong Kong’s Victoria Harbor, over 50% of shorelines have been armored, and the continuing growth of coastal immigration and urbanization is expected to increase the rate of shoreline armoring (Davis, Levin & Walther, 2002; Dugan et al., 2008; Lam, Huang & Chan, 2009; Patrick, Weller & Ryder, 2016). In the United States alone, about 14% of the lower 48 states’ shorelines are armored, and 64% of these armored shorelines are adjacent to estuaries and coastal rivers (Gittman et al., 2015).

Armored shorelines overall are associated with lower biodiversity, vegetation cover, and abundances of invertebrate and fish species (Moreira, Chapman & Underwood, 2006; Bilkovic & Roggero, 2008; Peterson et al., 2000; Firth et al., 2016a; Dugan et al., 2008; Morley, Toft & Hanson, 2012; Peters, Yeager & Layman, 2015; Gittman et al., 2016a). Armored shorelines can accelerate beach erosion as waves are reflected from armored structures (Heatherington & Bishop, 2012; Gittman et al., 2016b). They can reduce the overall ecological health of coastal ecosystems by degrading shallow intertidal habitats valuable for survival of juvenile fish and aquatic invertebrates (Bilkovic & Roggero, 2008; Seitz et al., 2006; Gittman et al., 2016a). Armored shorelines can also disrupt the transition between terrestrial and aquatic habitats as the gradual shoreline slope is abruptly steepened, which in turn can result in reduction of salt marsh habitats and submerged aquatic vegetation. Similarly, armoring also reduces deposition of woody debris and “wrack” or organic matter deposition on shorelines. This loss of organic debris can affect the aquatic–terrestrial food web, including fishes, macroinvertebrates associated with wrack and vegetated habitats, and birds (Bozek & Burdick, 2005; Dugan et al., 2008; Heerhartz et al., 2014; Harris, Strayer & Findlay, 2014; Heerhartz & Toft, 2015; Dethier et al., 2016; Wensink & Tiegs, 2016).

Recently, alternatives to shoreline armoring, including armoring removal, have emerged to simultaneously protect coastal urban infrastructure and restore ecological health (Davis et al., 2015; Gittman et al., 2016a; Bilkovic et al., 2017). Approaches include living shoreline techniques such as the creation of marsh sills in lieu of armoring shorelines in North Carolina (Bilkovic & Mitchell, 2013), restoring oyster reefs in Chesapeake Bay (Lawless & Seitz, 2014), restoring red mangrove colonization on riprap revetments in Biscayne Bay, Florida (Peters, Yeager & Layman, 2015), and managed realignment in Europe (Esteves & Williams, 2017). Few studies have assessed the effectiveness of armoring removal on restoring coastal ecosystems, but they generally demonstrate that shorelines without armoring can host higher abundances and diversity across different taxonomic groups. For example, marsh sills have higher abundance and diversity of fish and bivalves in shorelines of North Carolina (Gittman et al., 2016a) and introducing native riparian vegetation and logs after armoring removal can facilitate rapid response of macroinvertebrate assemblages in shorelines of Puget Sound, Washington (Toft, Cordell & Armbrust, 2014).

In Puget Sound, Washington, USA, there has been recent momentum to restore armored shorelines through removal of armoring structures, nourishment of sediments, re-planting native riparian vegetation, and addition of logs and woody debris (Toft et al., 2013b; Toft, Cordell & Armbrust, 2014). Such restoration efforts are driven by the need to protect Pacific salmon species such as endangered populations of Chinook salmon (*Oncorhynchus tshawytscha*) that are of cultural, ecological, and economic importance to the region (Rhodes et al., 2006; Munsch, Cordell & Toft, 2015). Juveniles of these and other salmon species use shallow intertidal areas as nursery habitats (Munsch, Cordell & Toft, 2016). Macroinvertebrate prey, both aquatic and terrestrial, are a vital part of Chinook diets and coastal food webs. Changes in their populations can negatively impact food availability for many fish species (Sobocinski, Cordell & Simenstad, 2010). Therefore, it is essential to ask whether shoreline restoration is having its intended effect, and to date, relatively little such analysis has been done.

Here we present an analysis of the effects of shoreline restoration, with the objective to determine how coastal biota respond when shoreline armor is removed, sediments nourished, and native vegetation planted. We assess responses across (a) study sites, (b) coastal biota type, (c) shoreline elevations, and (d) trajectories in time. Understanding these post-restoration dynamics can provide knowledge about factors that contribute to biological recovery that can be useful along other armored shorelines regionally and globally.

## Materials and Methods

### Study sites and sources of data

Puget Sound is a fjordal estuarine ecosystem comprising the southern part of the Salish Sea, which encompasses over 30,000 km^2^ in the Pacific Northwest, overlapping Washington, USA and British Columbia, Canada. This ecosystem comprises cold-temperate waters, river deltas, and shorelines mainly composed of clay, sand, mud, and gravel sediments originating from receded glaciers. Continued erosion of coastal bluffs contributes this sediment mix to Puget Sound beaches (Shipman, 2001). More than a quarter of the 4,000 km of shorelines in Puget Sound are armored (Puget Sound Partnership, 2016).

**Figure 1:**
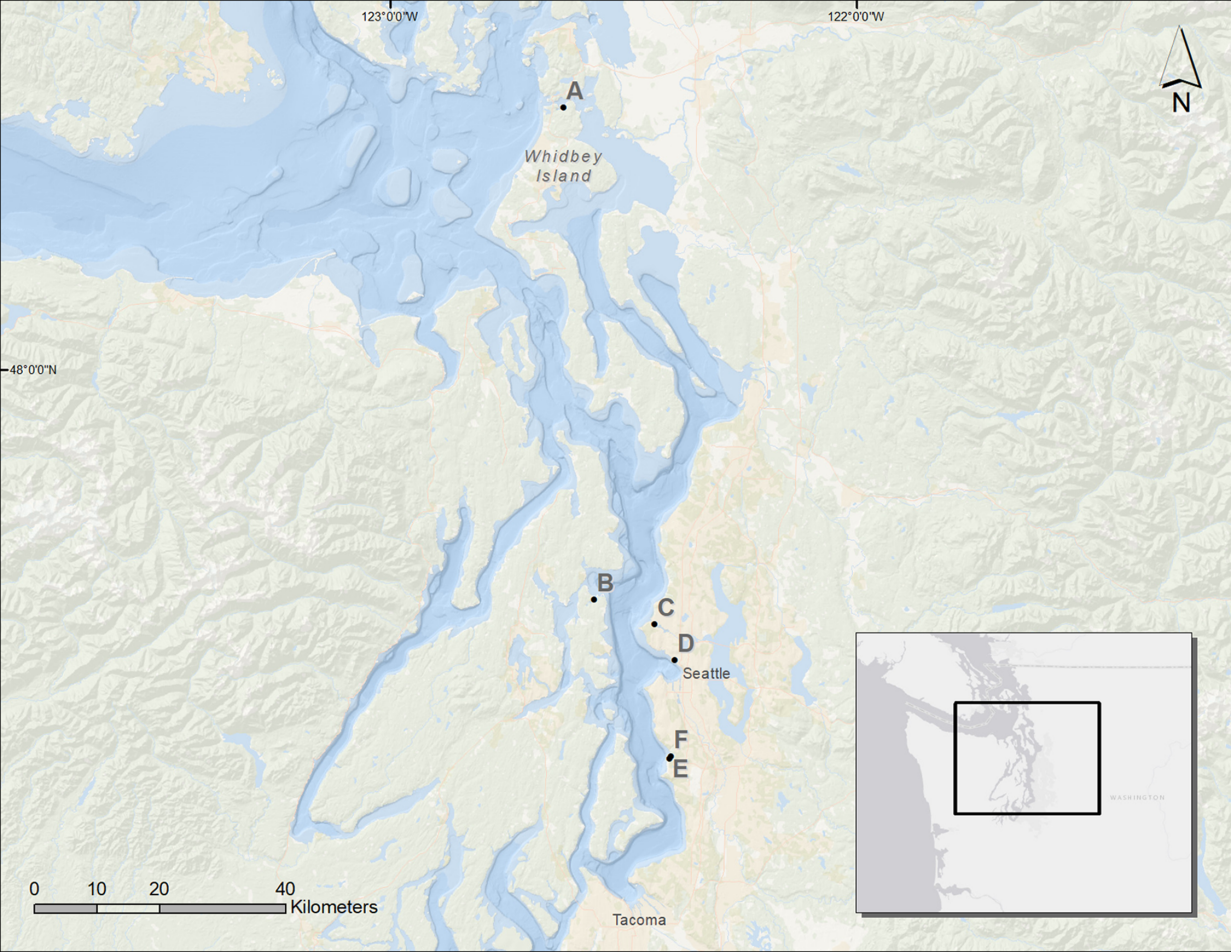
Map of the Puget Sound and the restored sites used for analysis. A, Cornet Bay; B, Powel Property; C, Salmon Bay Natural Area; D, Olympic Sculpture Park; E, Seahurst Park I (restored 2005); F, Seahurst Park II (restored 2014). Map Background: ArcGIS 10.2 Ocean Basemap (Credits: Esri, DeLorme, GEBCO, NOAA NGDC, and other contributors). Inset Map Background: ArcGIS Light Gray Canvas Map (Copyright: ©2013 Esri, DeLorme, NAVTEQ).

**Figure 2:**
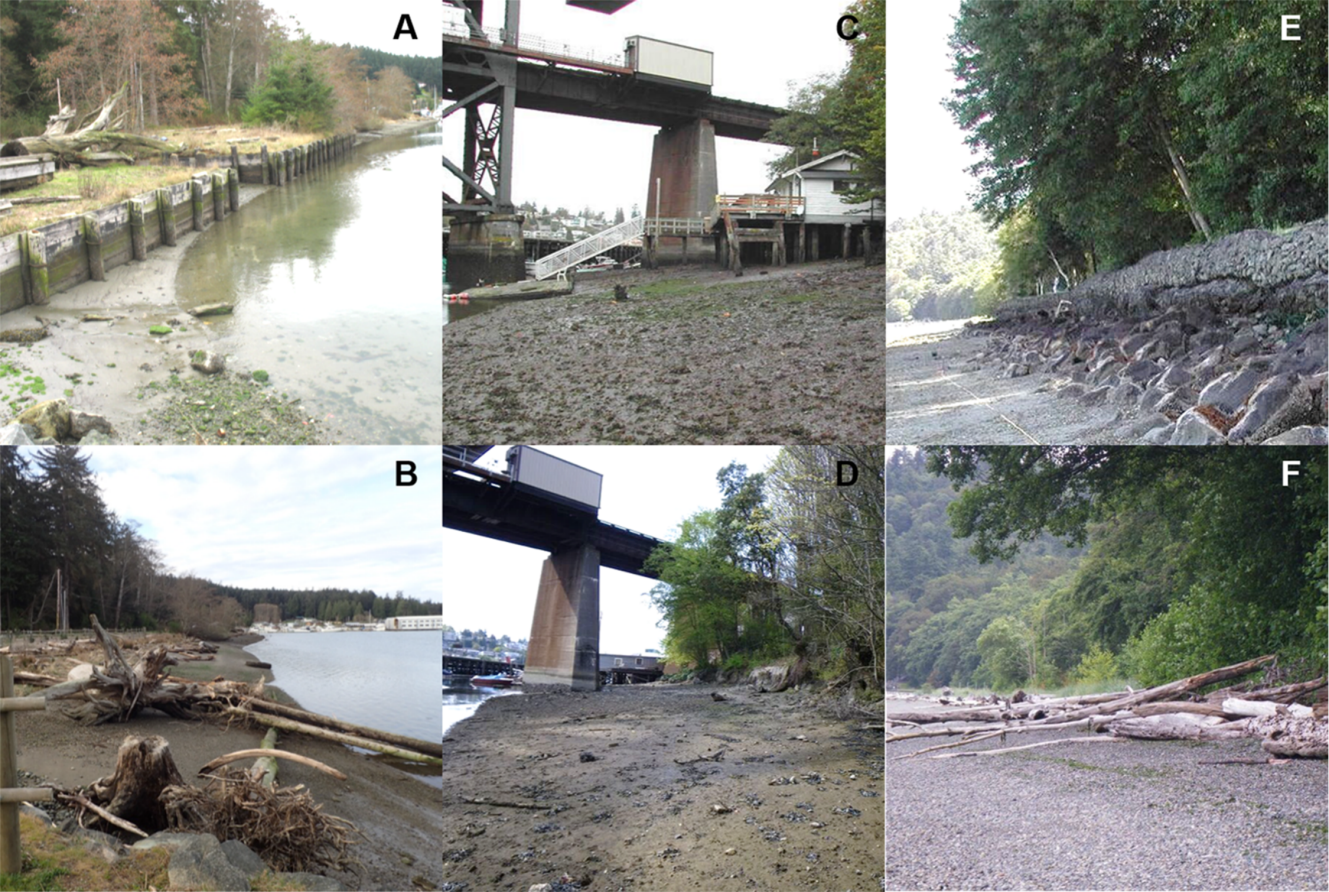
Three of the six restored sites used for this analysis. Frames (A, C, and E) show shorelines armored prior to their respective restorations and frames (B, D, and F) show shorelines in their restored state. Left to right: Cornet Bay (A, B), Salmon Bay Natural Area (C, D), Seahurst Park I (E, F). Photo Credit for frame (A): Sarah Schmidt. Photo Credit for frame (B): Lisa Kauman. Photo Credit for frames (C–F): Jason D. Toft.

We assessed six sites in Puget Sound, along which armoring was removed, to determine coastal biota responses (Fig. 1). These restored sites from north to south were Cornet Bay of Deception State Park on Whidbey Island, Powel Property in Port Madison on the north side of Bainbridge Island, Salmon Bay Natural Area which is a park downstream from the Hiram M. Chittenden Locks of Seattle, Olympic Sculpture Park in downtown Seattle, and two locations in Seahurst Park in the city of Burien, WA (Fig. 1; Table 1). All sites were formerly armored with concrete or wooden bulkhead and riprap, and the Salmon Bay Natural Area also had an overwater structure (Fig. 2). These sites were restored from 2005 to 2014 (Table 1), and monitoring was accomplished opportunistically depending on the year that sites were restored and the capacity for monitoring that was available. All data analyzed herein were compiled from site-specific reports, as stated in Table 1. Our analyses are new, building from previous technical reports and publications that represent individual sites, to increase spatial and temporal scales including the most recent post-restoration data.

**Table 1:** Sites used for analysis listed by pre-restoration year monitored (PR year), restoration year (Rest. year), post-restoration monitoring years and the types of coastal biota monitored.

Site	PR year	Rest. year	Post-restoration monitoring year							Coastal biota monitored					Elev.	Reference
			<1	1	2	3	4	5	10	W %	L #	SM %	MIC	MIR		
CB	2012	2013	–	X	–	–	–	–	–	X	X	–	X	X	–	Dethier et al. (2016)
PP	2012	2012	–	X	X	–	–	–	–	–	–	X	–	X	X	Adams, Padgham & Toft (2015)
SBNA	2004	2010	X	–	X	–	–	–	–	–	–	–	X	X	–	Toft et al. (2013a)
OSP	2005	2006	–	X	–	X	–	X	–	–	–	–	X	X	–	Toft et al. (2013b) and Cordell et al. (2017)
SHP I	2004	2005	–	X	–	X	–	X	X	–	–	–	X	X	X	Toft, Cordell & Armbrust (2014) and Toft (2016)
SHP II	2010	2014	–	X	–	–	–	–	–	–	–	–	X	X	X	Toft (2016)

**Notes:**

CB, Cornet Bay; PP, Powel Property; SBNA, Salmon Bay Natural Area; OSP, Olympic Sculpture Park; SHP I, Seahurst Park restored in 2005; SHP II, Seahurst Park Restored in 2014; W %, wrack % cover; L #, number of logs; SM %, saltmarsh % cover; MIC, macroinvertebrate counts; MIR, macroinvertebrate richness; Elev., shoreline elevation sampling.

Five ecosystem metrics were monitored before and after restoration, with one site monitored up to 10 years after restoration (Table 1). Survey data varied by site due to goals and characteristics of each restoration site, and included counts and richness (number of individual taxa) of macroinvertebrates for both terrestrial and aquatic groups, wrack % cover, number of logs (which have both biotic and physical attributes), and saltmarsh % cover. Three sites were also monitored at two different shoreline elevations (Table 1).

While we sampled macroinvertebrates at all six sites, we also sampled non-macroinvertebrate ecosystem metrics at two sites, Cornet Bay and Powel Property (Table 1). At Cornet Bay, we sampled five replicates of terrestrial and aquatic macroinvertebrates before restoration (July 2012) and after restoration (July 2013) across a 50 m transect parallel to the shore (Dethier et al., 2016). During those same respective days, we also sampled 10 replicates of total wrack % cover and counted five replicates of total number of logs before and after restoration (Dethier et al., 2016). We collected wrack samples and the top 2.5 cm of sediment layer using a 15 cm diameter benthic corer; all invertebrates were separated from the wrack and counted and identified to the lowest possible taxonomic classification using a dissecting microscope (Dethier et al., 2016). At Powel Property, we sampled three replicates of macroinvertebrates for one year before restoration (2012) and two years after restoration (2013 and 2014). This sampling design was replicated across low (3.05 m mean lower low water or MLLW) and high (3.66 m MLLW) shoreline elevations (Adams, Padgham & Toft, 2015) corresponding to the placement of the armoring. In addition, we also sampled for saltmarsh % cover across three replicates for the same pre- and post-restoration years monitored for macroinvertebrates.

For the other four sites, we used macroinvertebrate counts and richness metrics for both aquatic and terrestrial types (Toft et al., 2013a, 2013b; Toft, Cordell & Armbrust, 2014; Toft, 2016; Cordell et al., 2017). At Salmon Bay Natural Area, we sampled seven replicates of aquatic invertebrates using sediment cores and terrestrial insects using plastic bin fall traps for one year before restoration (2004) and two post-restoration years (2010 and 2012) across April, May, June, and July (Toft et al., 2013a). We repeated this sampling approach in two shoreline elevations, 0.3 and 2.44 m MLLW. At Olympic Sculpture Park, we sampled seven replicates of aquatic invertebrates using an epibenthic pump that sampled the water–sediment interface, and terrestrial insects applying the same sampling gear used in the Salmon Bay Natural Area for one year before restoration (2005) and three post-restoration years (2007, 2009, 2011) across April, May, June, and July (Toft et al., 2013b). In Seahurst Park, we sampled two shorelines, one restored in 2005 and the other restored in 2014 (Table 1). For both the 2005 and 2014 restored shorelines, we sampled aquatic and terrestrial macroinvertebrates using the same sediment core and fallout trap sampling gear as the previous sites (Toft, Cordell & Armbrust, 2014; Toft, 2016). In the 2005 restored shoreline, we sampled seven replicates of one year before restoration (2004) and four separate years after restoration (2006, 2008, 2010, 2015) across the months of June, July, and September (Toft, 2016). Our sampling design for the 2014 restored shoreline was the same, except that we monitored one year before restoration in 2010 and one year after restoration in 2015 (Toft, 2016).

### Quantitative analysis

To measure the effectiveness of shoreline restoration on coastal biota, we used Cohen’s *D* Effect Size (Cohen, 1992). This effect-size statistic has been widely used, for example to measure effects of stream engineering on increasing salmon abundances, pine forest restoration on native understory vegetation, and invasive vegetation removal on restoring native woody plants (Taylor, Smith & Haukos, 2006; McGlone, Springer & Laughlin, 2009; Stewart et al., 2009). Cohen’s *D* is calculated with the following equation:

$$D = {{\left( {{{\rm{\mu }}_{\rm{A}}} - {{\rm{\mu }}_{\rm{B}}}} \right)} \over {\rm{\sigma }}}$$

where μ_A_ is the mean value of measured variable (e.g., counts, richness, percent cover) after restoration, μ_B_ is the mean value of measured variable before restoration, and σ is the pooled standard deviation. Although values of *D* are likely to vary with context, as a general guideline, when *D* less than 0.2, the restoration is considered to have had no effect, while 0.2–0.8 indicate moderate effect, and 0.8 or greater indicates substantial effect (Rosnow, Rosenthal & Rubin, 2000). In our analysis, the greater the values of *D*, the greater positive response to restoration, in contrast to the armored state and presumably more toward a pre-disturbance natural state.

We calculated *D* for restoration effects in the following four major categories: (1) separately for each study site, (2) specific to each monitored type of coastal biota, (3) at the two elevations of the base and placement of armoring, and (4) trajectory in time of post-restoration years. For the study sites category, we calculated the effect sizes of all the respective coastal biota monitored for each site individually. For the monitored coastal biota, we calculated the effect sizes for the five types of coastal biota: wrack % cover, number of logs, saltmarsh cover, macroinvertebrate counts, and macroinvertebrate richness. For the shoreline elevation, we calculated effect sizes for the elevation at the base of the armoring and at the elevation where armoring formerly stood. Lastly, for the trajectory in time we calculated effect sizes for the six post-restoration monitoring years. To test for statistical significance of *D*, we performed one-sample two-tailed *t*-tests (α = 0.05), comparing the observed data against the null hypothesis (*H*_0_: μ = 0). Where comparing the means of two different elevations, we used a two-sample two-tailed *t*-test.

## Results

**Figure 3:**
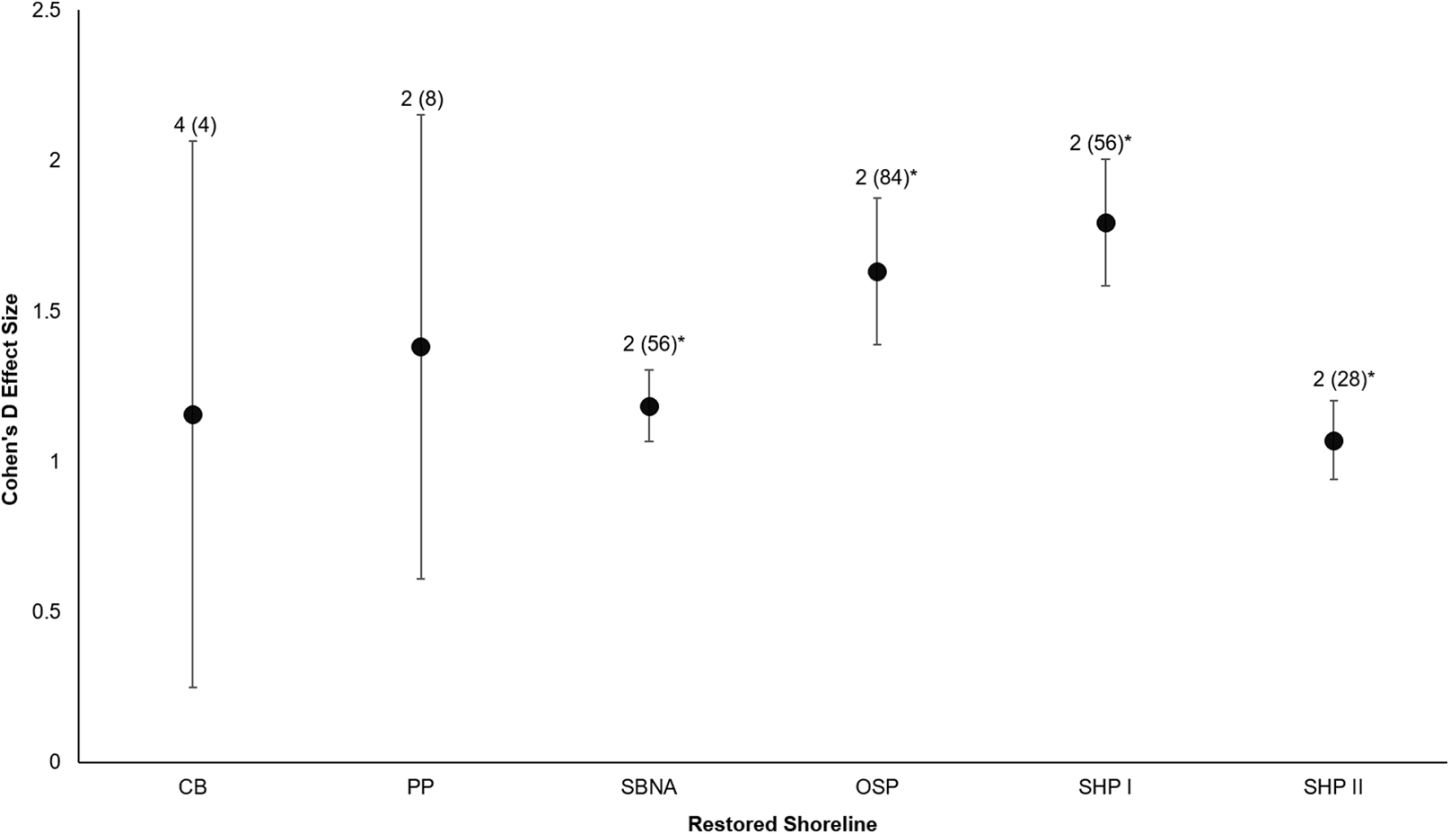
Cohen’s *D* Effect Sizes (±SE for error bars) across six restored sites of Puget Sound used in the analysis (site acronyms same as in Table 1). Data labels show the number of coastal biota types monitored and the sample sizes (the number of effect sizes for each site). Effect sizes with asterisks were significantly different from zero.

**Figure 4:**
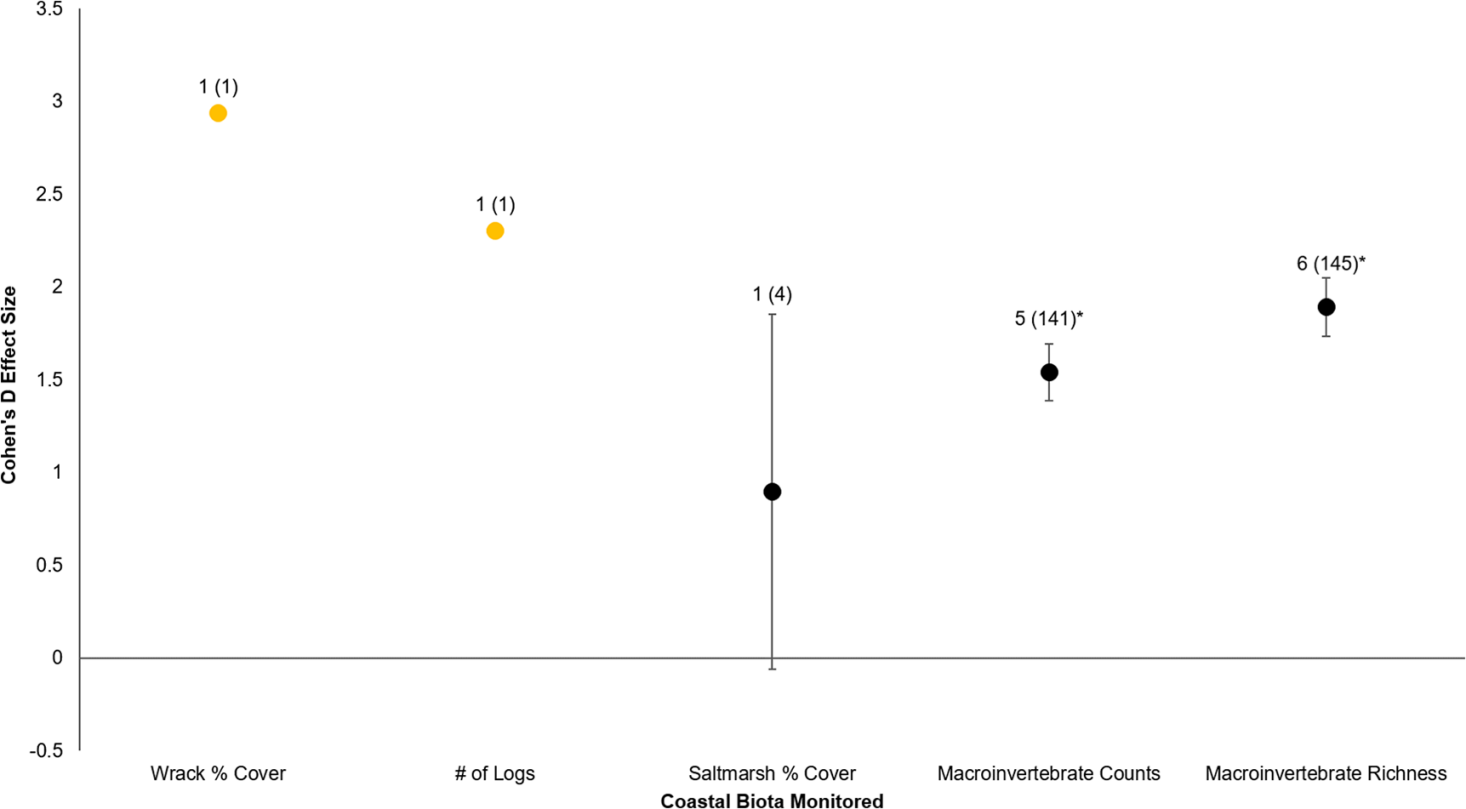
Cohen’s *D* Effect Sizes (±SE for error bars) by five major types of coastal biota monitored. Data labels show number of restored sites that were monitored and the sample sizes (the number of effect sizes for each biota type). Coastal biota labeled in orange were not integrated for individual *t*-tests due to lack of replicates. Coastal biota effect sizes with asterisks were significantly different from zero.

**Figure 5:**
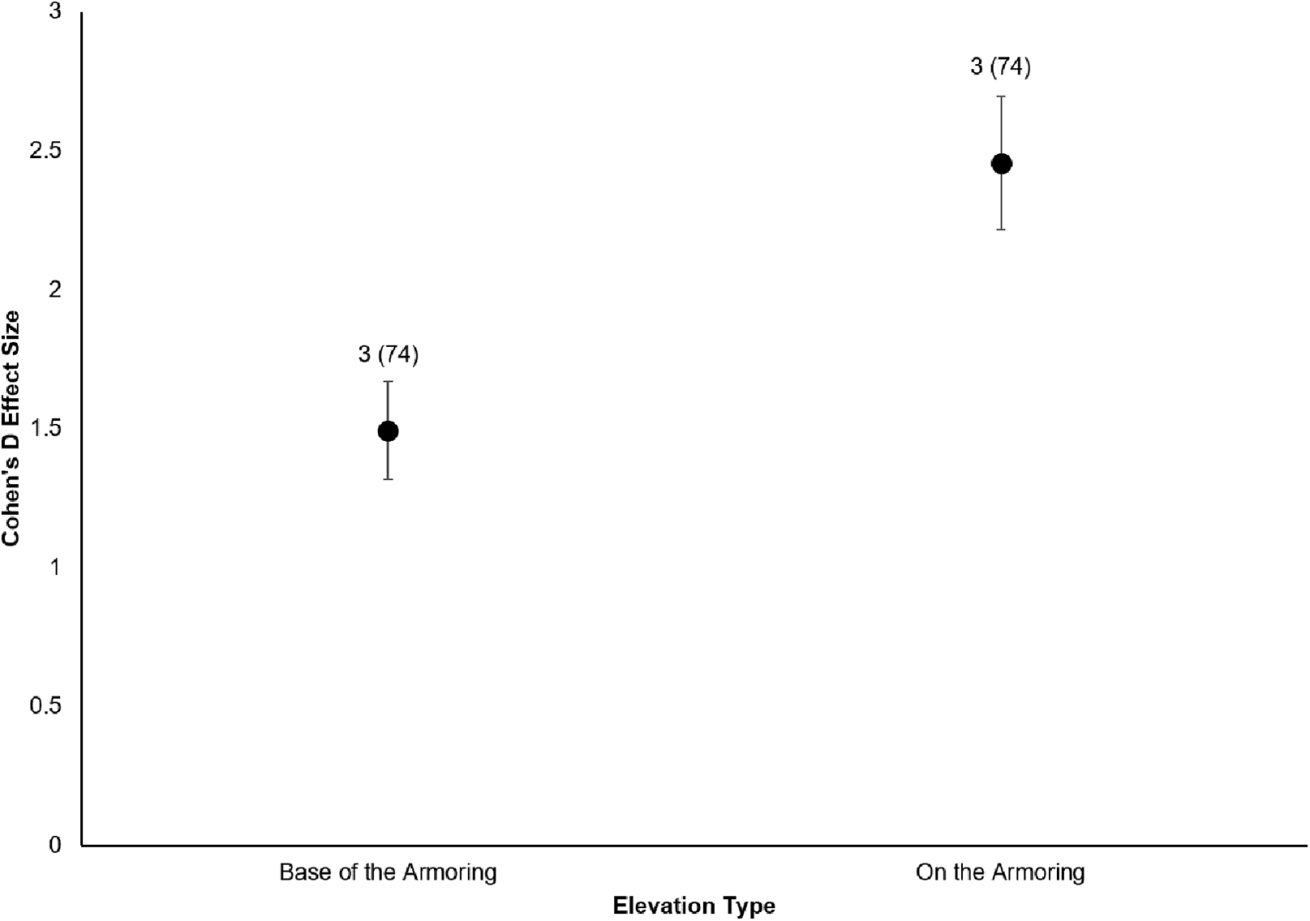
Cohen’s *D* Effect Sizes (±SE for error bars) by elevation monitored. Data labels show the number of restored sites and the sample sizes (the number of effect sizes for each elevation type). Both elevations’ effect sizes were significantly different from zero.

All six sites demonstrated positive responses with the mean effect size varying between 1.07 and 1.79 (Fig. 3). Four of the six sites had statistically significant responses (Fig. 3). These four sites were the Salmon Bay Natural Area, Olympic Sculpture Park, and the two Seahurst Park sites (Salmon Bay: *t*_0.05(2),54_ = 9.9, *p* < 0.001, 95% CI = 0.95, 1.43; Olympic Sculpture Park: *t*_0.05(2),83_ = 6.69, *p* < 0.001, 95% CI: 1.14, 2.12; Seahurst Park Restored 2005: *t*_0.05(2),55_ = 8.49, *p* < 0.001, 95% CI = 1.37, 2.22; Seahurst Park Restored 2014: *t*_0.05(2),27_ = 8.23, *p* < 0.001, 95% CI: 0.8, 1.34). Among sites and elevations, all five coastal biotic measures showed similarly positive effects, and two of the three that could be analyzed with *t*-tests were significant (Figs. 4 and 5). These two biotic measures were macroinvertebrate counts and richness (Macroinvertebrate Counts: *t*_0.05(2),140_ = 10, *p* < 0.001, 95% CI: 1.23, 1.84; Macroinvertebrate Richness: *t*_0.05(2),144_ = 12.01, *p* < 0.001, 95% CI: 1.58, 2.20). Mean effect size was stronger in higher shoreline elevations where armoring had previously been directly placed (Base of the Armoring: *t*_0.05(2),73_ = 8.44, *p* < 0.001, 95% CI: 1.14, 1.85; On the Armoring: *t*_0.05(2),73_ = 10.24, *p* < 0.001, 95% CI: 1.98, 2.93).

**Figure 6:**
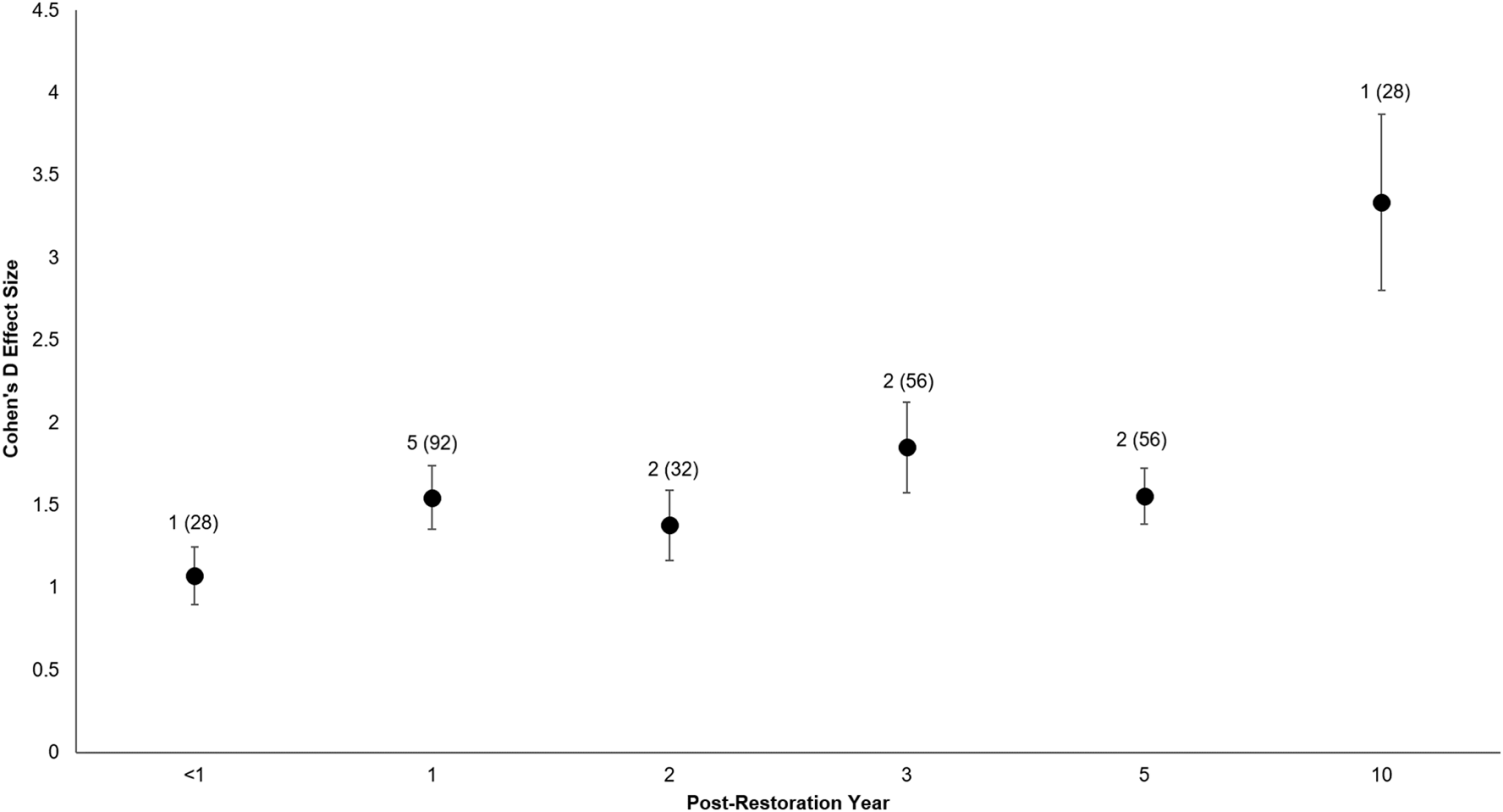
Cohen’s *D* Effect Sizes (±SE) by post-restoration years monitored. Effect sizes reflect comparisons between pre-restoration and post-restoration for each respective post-restoration year monitored. Data labels show the number of restored sites and sample sizes (the number of effect sizes for each post-restoration year monitored). Effect sizes of all post-restoration years were significantly different from zero.

All of the post-restoration years demonstrated positive and statistically significant responses (*p* < 0.001 for all post-restoration years), with year 10 showing the greatest positive response ($\bar X = 3.34$) and year <1 showing the lowest ($\bar X = 1.07$) (Year <1: *t*_0.05(2),27_ = 6.07, *p* < 0.001, 95% CI: 0.71, 1.43; Year 1: *t*_0.05(2),91_ = 8.02, *p* < 0.001, 95% CI: 1.16, 1.93; Year 2: *t*_0.05(2),31_ = 6.5, *p* < 0.001, 95% CI: 0.95, 1.81; Year 3: *t*_0.05(2),55_ = 6.71, *p* < 0.001, 95% CI: 1.30, 2.40; Year 5: *t*_0.05(2),55_ = 9.2, *p* < 0.001, 95% CI: 1.22, 1.89; Year 10: *t*_0.05(2),27_ = 6.25, *p* < 0.001, 95% CI: 2.24, 4.43) (Fig. 6).

## Discussion

Armoring removal in Puget Sound has resulted in diverse, positive responses by coastal biota. All four major effect categories (restored sites, coastal biota, shoreline elevation, and trajectory in time) showed substantially positive responses (effect size >0.8; Rosnow, Rosenthal & Rubin, 2000). Furthermore, there was clear evidence that coastal biota responded quickly (within a year after restoration), with subsequent post-restoration years maintaining or even increasing biotic and abiotic gains. Of the five major types of coastal biota, recovery was strongest for macroinvertebrates; the responses of wrack cover, number of logs, and saltmarsh cover were also positive but they had smaller sample sizes and therefore had weaker statistical inferences.

The strong significant responses of macroinvertebrates were consistent with previous work on restoration of individual marine sites (Toft et al., 2013b; Toft, Cordell & Armbrust, 2014) and other aquatic habitats, most of which are in freshwater ecosystems. Some macroinvertebrate restoration response studies come from rivers and channelized streams, where restoring some habitat complexity results in greater abundances and diversity of macroinvertebrates (Korsu, 2004; Muotka & Syrjänen, 2007; Miller, Budy & Schmidt, 2010). Wetlands that have been restored or newly created can be quickly colonized by macroinvertebrates, especially those with greater dispersal capability such as aerial insects (Brown, Smith & Batzer, 1997; Stewart & Downing, 2008). Our analysis similarly showed that overall, macroinvertebrate responses to restoration in coastal ecosystems are positive and substantial, which in turn can enhance prey availability for migratory fishes and seabirds and improve ecosystem health as a whole (Dugan et al., 2003; Heerhartz & Toft, 2015).

With regards to pace of response, we found that coastal biota can quickly and positively respond to shoreline armor removal and that this trend can continue across multiple post-restoration years. Biotic recovery can be similarly rapid after restoration of channelized streams, saltmarsh habitats, and oyster reefs (Warren et al., 2002; Miller, Budy & Schmidt, 2010; La Peyre et al., 2014). However, this is not always the case, because abundances and diversity of macroinvertebrates can initially drop significantly after restoration of a channelized stream (Muotka & Syrjänen, 2007; Korsu, 2004). Muotka & Syrjänen (2007) found that macroinvertebrate recovery was not pronounced until four to six years, but the variability appeared to stabilize after eight years. Biotic recoveries in restored wetlands were also slow when compared with reference sites across the same temporal scales, and the richness of invertebrates and fishes did not approach their peak trajectories until three to five years after restoration (Simenstad & Thom, 1996; Morgan & Short, 2002). Similar perceptions have been recorded for seagrasses, as restoration shows a greater response when monitored for seven versus three years (Bell, Middlebrooks & Hall, 2014). While our sites experienced rapid recovery, the same might not hold for other similar coastal restoration projects outside the Puget Sound. As is the case with restored wetlands, restored coastlines may not replace the full natural functions of healthy coastlines, and may take longer than their respective monitoring periods to reach fully self-sustaining ecosystem functioning status (Zedler & Callaway, 1999).

We also found that coastal biota directly affected by armoring placement (higher shoreline elevations) responded more positively than biota that were indirectly affected (lower shoreline elevations). Toft, Cordell & Armbrust (2014) came to similar conclusions, with less response seen in biota below the footprint of armoring. This is an interesting comparison to eco-engineering of armoring, where diversity can be higher at lower elevations (Firth et al., 2016b), contrasting the expectations from hard versus soft restoration approaches. In rivers, biota within sections directly affected by dam impoundment can respond positively to dam removal within days to a few years; these biotic parameters include but are not limited to recolonization by native riparian plants and lotic organisms (Hart et al., 2002). Upstream areas that were indirectly affected by dam impoundment may not experience full recovery of aquatic–terrestrial ecosystem linkages until years to decades after the dam removal (Hart et al., 2002). In contrast, previous work on ecological responses to removal of dikes in estuarine tidal wetlands found that habitats indirectly affected by dikes are just as likely to respond positively during a similar temporal scale to habitats directly affected in terms of marsh vegetation, benthic macroinvertebrates, fish, and other megafauna (Hood, 2004).

One of the challenges in our analysis was the small sample size in certain parameters. Quantified effect responses of wrack % cover, saltmarsh % cover, and number of logs were weaker than for parameters with larger sample sizes. Small sample sizes can increase error rate and potentially distort response interpretations (Raudys & Jain, 1991), so we cannot necessarily generalize our results to other shorelines restored through similar means. However, it is important to note that wrack % cover can increase abundances and diversity of macroinvertebrates, facilitate saltmarsh growth, and support megafauna such as seabirds (Dugan et al., 2003; Chapman & Roberts, 2004; Smith, 2007; Harris, Strayer & Findlay, 2014; Heerhartz et al., 2014). Natural shorelines have higher woody debris counts and densities than armored shorelines, and these can enhance and retain wrack % cover but also reduce beach erosion (Angradi et al., 2004; Eamer & Walker, 2010; Harris, Strayer & Findlay, 2014; Heerhartz et al., 2014). Based on the suspected functions that wrack, saltmarsh, and logs provide in healthy coastlines, it is essential to increase the geographical scope and number of studies of these coastal biota types to assess the successful recovery of restored coastal ecosystems. Related to this is the added benefit that before–after control-impact (BACI) techniques would have in aiding interpretation (Underwood, 1994), emphasizing that increased sample size of restored and reference sites over time will be essential to fully understand restoration effectiveness.

While our sample size at the site scale was limited to six, this opportunistic sampling can be advantageous to efficiently produce information-rich results (Palinkas et al., 2015). Opportunistic sampling can also detect presence of certain ecosystem metrics in regions where systematic sampling would not detect them (De Barba et al., 2009). As our results have now demonstrated that armoring removal can elicit rapid ecosystem recovery, and site-specific studies that use BACI analysis have shown similar results (Toft et al., 2013b; Toft, Cordell & Armbrust, 2014), applying BACI in future monitoring studies will be helpful to increase the scale of inference. Furthermore, continuing to monitor the metrics through additional post-restoration years and expanding the pre- and post-restoration monitoring approach to other shorelines may reduce bias caused by opportunistic sampling, such as unequal sample sizes and preferential sampling (De Barba et al., 2009). This will be essential to expand the temporal scale, as most of our sites were restored for less than five years, with only one site restored for 10 years.

Coastal biota recovery after armoring removal may also be hindered or facilitated by abiotic variables and their responses to restoration, which we did not address in our analysis. Beach profiles and sediment grain size may change slowly in response to the placement of shoreline armoring (Dethier et al., 2016), which suggests that these two variables may likewise experience slow recovery across seasons to years after armoring removal. Furthermore, sediment changes following armor removal, such as increasing or decreasing deposition of fines, likely affect recovery patterns of biota. Lower shoreline elevations are also more susceptible to disturbances by hydrological and oceanographic processes, which in turn may prevent rapid recovery of associated coastal biota (Harris, Strayer & Findlay, 2014).

The responses of coastal biota observed in our analysis may be attributed to “passive” ecosystem management post-recovery. The passive approach does not require further action to be taken after a restoration project is completed (Simenstad, Reed & Ford, 2006). Instead, it allows disturbances to occur, potentially influencing the recovery trajectory of ecosystem components. Shorelines are exposed to frequent disturbances such as waves, flooding, and storm-related events (Gittman et al., 2016b). This can enhance recovery by depositing wrack along shorelines, providing habitats for grazing arthropods and saltmarsh vegetation (Dugan et al., 2003; Chapman & Roberts, 2004). Reestablishing the natural cycle of disturbance and recovery driven by various oceanographic processes has the potential to elicit positive responses by coastal biota for years after initial restoration.

## Conclusion

Here we have shown that biotic metrics can respond strongly and positively to armor removal and restoration of beaches. Even with our pronounced results, reversing shoreline armoring is and will continue to be a management challenge. Coastal habitats around the world face unprecedented urban growth (Gittman et al., 2015). In the United States, shoreline armoring is primarily driven by development of residential properties, attempts to improve domestic and international shipping traffic, and protection against storm events (Gittman et al., 2015). With nearly half of the world’s population expected to live within 100 km from shorelines by 2030, it is safe to assume that armoring will continue to increase within and outside the United States in the next few decades (MEA, 2005; Gittman et al., 2015, 2016b). However, it is critical to recognize that shorelines without armoring can function as natural erosional barriers. For example, large woody debris protects from beach erosion but also enhances wrack accumulation, which in turn can enhance saltmarsh growth and improve aquatic–terrestrial connectivity (Chapman & Roberts, 2004; Eamer & Walker, 2010; Heerhartz et al., 2014). Removing shoreline armoring and improving aquatic–terrestrial connectivity is not only beneficial to the ecosystem but also can help coastal communities and livelihoods (Firth et al., 2016b), because ecosystem components that are harvested (such as fishes) rely on ample availability of macroinvertebrate prey for survival.

It is therefore critical for policymakers to consider numerous benefits of shoreline armoring removal before undertaking new shoreline development. While removal of armoring is not feasible in all cases due to financial or safety concerns, it is clear from this study that restoring shorelines through armoring removal can potentially benefit coastal ecosystem health and coastal populations by increasing ecosystem services. Furthermore, many shoreline homeowners are increasingly recognizing environmental impacts of shoreline armoring and express a preference for natural shoreline structures, as they can be aesthetically appealing and have many ecological benefits (Scyphers, Picou & Powers, 2014). Existing and new shoreline management policies should encourage homeowners and other stakeholders to protect natural shorelines and embrace shoreline restoration when it can simultaneously protect properties, coastal populations, biodiversity, and retain ecosystem services.

## Supplemental Information

10.7717/peerj.4275/supp-1 Supplemental Information 1 Cornet Bay invertebrate data. Dataset of invertebrates sampled in Cornet Bay before and after shoreline armoring removal.

10.7717/peerj.4275/supp-2 Supplemental Information 2 Cornet Bay wrack cover and log data. Dataset of wrack cover and number of logs in Cornet Bay sampled before and after shoreline armoring removal.

10.7717/peerj.4275/supp-3 Supplemental Information 3 Powel Property dataset. Dataset of saltmarsh percentage cover and invertebrate richness from Powel Property before and after shoreline armoring removal.

10.7717/peerj.4275/supp-4 Supplemental Information 4 Salmon Bay Natural Area invertebrates. Dataset of macroinvertebrates sampled in Salmon Bay Natural Area before and after armoring removal.

10.7717/peerj.4275/supp-5 Supplemental Information 5 Epibenthos data from Olympic Sculpture Park. Dataset of epibenthos sampled in Olympic Sculpture Park before and after shoreline armoring removal.

10.7717/peerj.4275/supp-6 Supplemental Information 6 Olympic Sculpture Park insect data. Dataset of insects sampled before and after shoreline armoring removal in Olympic Sculpture Park.

10.7717/peerj.4275/supp-7 Supplemental Information 7 Seahurst Park invertebrate dataset. Dataset of invertebrates sampled before and after shoreline armoring removal at two different locations (removed in 2005 and 2014 respectively) in Seahurst Park.

## Additional Information and Declarations

### Data Availability

The following information was supplied regarding data availability: The raw datasets have been provided as Supplemental Dataset Files.
